# Seraph® filter effectiveness in the treatment of circuit-related infections in ECMO patients—a single-center report

**DOI:** 10.3389/fmed.2025.1664552

**Published:** 2025-10-17

**Authors:** Daniel Lovric, Ivan Situm, Vanja Nedeljkovic, Mate Mogus, Ante Erceg, Mirabel Mazar, Slobodan Mihaljevic, Vedran Premuzic

**Affiliations:** ^1^Intensive and Acute Cardiac Care Unit, Department for Cardiovascular Diseases, University Hospital Center Zagreb, Zagreb, Croatia; ^2^School of Medicine, Catholic University of Zagreb, Zagreb, Croatia; ^3^Clinic of Anesthesiology, Resuscitation and Intensive Care, University Hospital Center Zagreb, Zagreb, Croatia; ^4^Special Hospital for Lung Diseases, Zagreb, Croatia; ^5^School of Medicine, University of Zagreb, Zagreb, Croatia; ^6^Department of Nephrology, Hypertension, Dialysis and Transplantation, University Hospital Center Zagreb, Zagreb, Croatia

**Keywords:** ECMO, hemadsorption, infection, Seraph 100, bacteria

## Abstract

**Introduction:**

Nosocomial infections in patients undergoing extracorporeal membrane oxygenation (ECMO) are common and associated with poorer outcomes. This study aimed to analyze the efficacy of the Seraph filter in reducing circuit-related infections and improving clinical outcomes in COVID-19 patients treated with ECMO.

**Methods:**

In 13 patients who required ECMO support, circuit-related infections were diagnosed based on either local signs of infection accompanied by positive microbiological samples taken locally or positive blood cultures. Hemoadsorption was performed using Seraph 100 in series with the dialyzer for 4–6 h. Blood cultures were also sampled both before and 24 h after treatment with Seraph 100.

**Results:**

Sterile blood cultures were achieved in 53.8% of all patients. Blood cultures were found to be sterile from fungi (*Candida parapsilosis/glabrata*) and *Stenotrophomonas malthophilia* in all patients. In addition, the blood cultures were sterile from *Klebsiella pneumoniae* in 60%, *Staphylococcus epidermidis in* 75%, and *Acinetobacter baumanii* in 50% of all patients. We observed a significant reduction in vasopressor doses, an improvement in respiratory parameters, and a significant decrease in the sequential organ failure assessment (SOFA) score on the first day after treatment with Seraph 100.

**Conclusion:**

This is the first study on ECMO patients treated with Seraph 100 for circuit-related infections. It shows a significant percentage of negative blood cultures after hemadsorption, particularly for fungal infections. These findings should be validated in multicenter prospective studies.

**Clinical trial registration:**

ClinicalTrials.gov, identifier NCT05470907.

## Introduction

Extracorporeal membrane oxygenation (ECMO) support provided to patients who need mechanical assistance due to respiratory and cardiocirculatory failure. These patients are at a high risk of mortality, especially if they develop septic shock (1, 2). Additionally, ECMO due to the artificial surfaces of the ECMO circuit, can provoke an inflammatory reaction and exacerbate the pre-existing pro-inflammatory state associated with sepsis (3, 4). The rates of nosocomial infections in patients on ECMO have been reported to be as high as 64% and are associated with poorer outcomes (5–8). Patients who require ECMO support for a prolonged duration frequently develop cannula-related infections (8, 9). Previous studies have reported the isolation of *Pseudomonas aeruginosa, Staphylococcus* spp., and *Enterobacteriaceae* in patients with cannula-related infections, while the reported incidence of fungal infection was very low (8–10). The risk factors associated with the incidence of cannula-related infections include a longer duration of ECMO, higher severity scores upon admission, and positive blood cultures (9, 10).

ECMO was used extensively during the COVID-19 pandemic in critically ill patients with acute respiratory distress syndrome (ARDS) whose condition did not improve with mechanical ventilatory support (11–13). Patients with COVID-19 who had prolonged ICU stays were especially prone to developing secondary bacterial co-infections, and their overall mortality rate was high (14–17). Co-infections may be exacerbated due to the usage of steroids and other immune-modulatory agents, such as IL-6 antagonists.

The Seraph 100® Microbind Affinity Blood Filter (Seraph 100; Exthera Medical Corporation, Martinez, CA) is an extracorporeal hemoadsorption device with a broad-spectrum sorbent capable of binding bacteria, viruses, and fungi in the blood, including SARS-CoV-2, through a heparin microbead-mediated mechanism (18–20).

Some studies have reported improvements in hemodynamics and inflammatory biomarkers after treatment with the Seraph filter (18, 21). However, there are only a few case reports that describe patients with septic shock on ECMO who also received Seraph therapy for its pathogen removal properties (22, 23). The aim of this study was to analyze the efficacy of the Seraph filter in critically ill ICU patients with COVID-19 on ECMO, specifically in reducing cannula-related infections and improving clinical outcomes. The study also sought to explore possible differences in reducing cannula-related infections between patients who underwent a complete ECMO system change and those who did not.

## Materials and methods

All patients with COVID-19 admitted to the ICU from January 2021 to February 2023 who received veno-arterial (VA) or veno-venous (VV) ECMO due to ARDS were included in this retrospective, single-center, observational study. As standard practice, SARS-CoV-2 infection was diagnosed using a real-time reverse transcription polymerase chain reaction (RT-PCR) test on nasal/oral swabs.

Cannulation was performed either percutaneously using the Seldinger technique or openly under direct vision. For VA-ECMO, all patients were cannulated via the femoral vein, with the inflow cannula positioned up to the level of the right atrium, and the ipsilateral femoral artery was used for the outflow cannula. All patients also had reperfusion cannulas placed into the superficial femoral artery, distal to the outflow cannula, to ensure sufficient distal circulation and prevent limb ischemia. In patients for whom ECMO therapy was successful, the cannulas were removed under sterile conditions. While patients were on ECMO support, blood cultures were routinely performed when infection was suspected and during any concerns of sepsis. Cannula-related infection was diagnosed based on either local signs of infection combined with positive microbiological samples taken locally or upon finding positive microbiological blood cultures, after potential sources of sepsis such as other intravascular catheters were exchanged. Blood cultures were sampled before and 24 h after treatment with the hemoadsorber Seraph 100.

**Figure 1:**
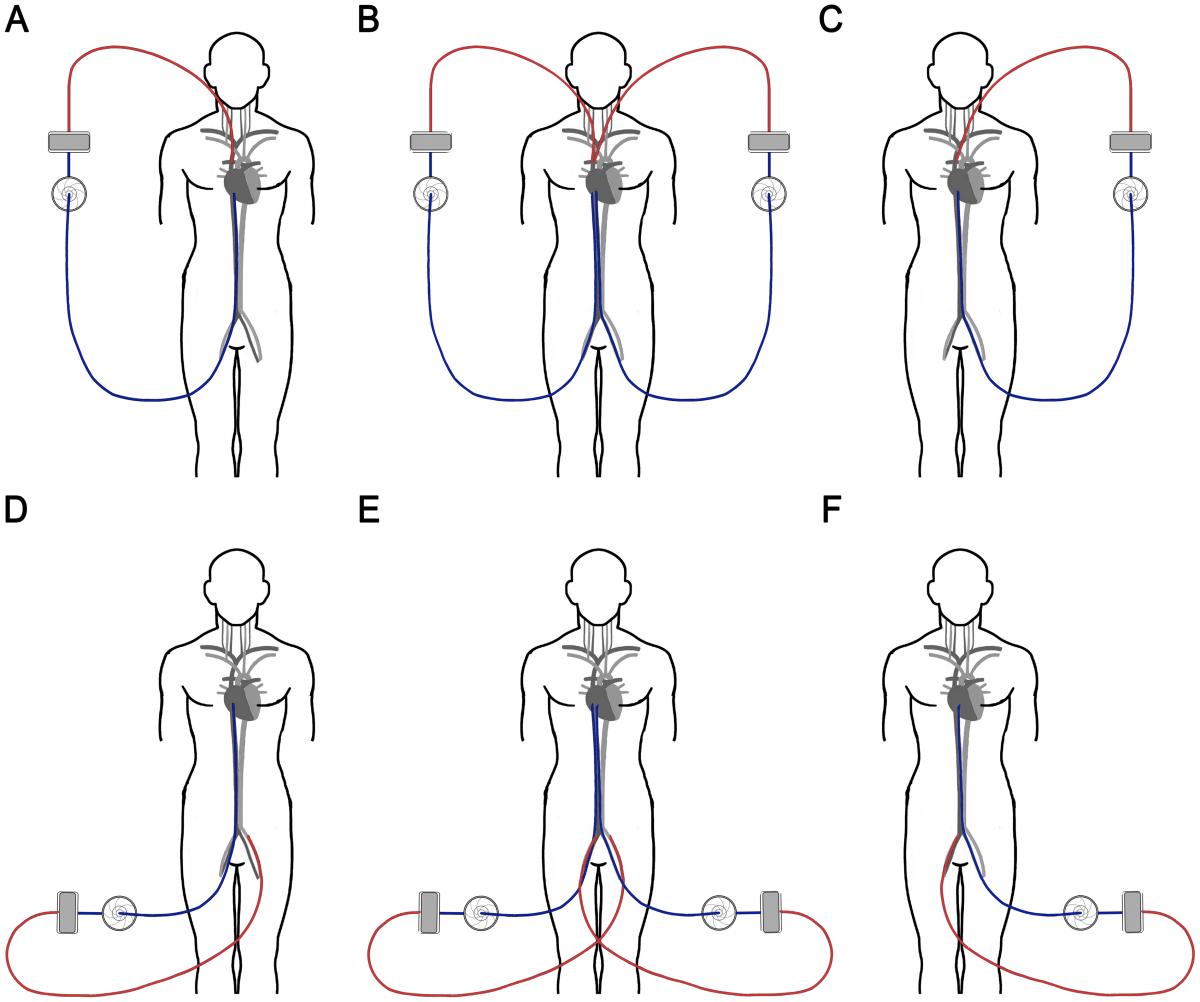
Schematic diagram of the ECMO circuit exchange. In VV-ECMO **(A)**, we first placed a new set of cannulas in the contralateral veins and started another ECMO circuit **(B)**, then we removed the original circuit and cannulas **(C)**. In VA-ECMO **(D)**, we placed a new arterial outflow cannula in the femoral artery on the side that previously contained the venous inflow cannula and a new inflow cannula in the femoral vein on the side where the arterial outflow cannula was placed **(E)**. Then, we removed the original circuit **(F)**. ECMO, Extracorporeal membrane oxygenation; VV, veno-venous; VA, veno-arterial.

In accordance with local institutional guidelines, hemoadsorption was performed using Seraph 100 (Exthera Medical Corporation, Martinez, CA) on all COVID-19 patients receiving ECMO who were suspected of having cannula-related sepsis. In five patients, the entire ECMO system was exchanged, including the cannulas, by first placing the new system in parallel, since the patients were too unstable to have the old system removed beforehand (Figure 1). These patients underwent two Seraph 100 procedures—one prior to the ECMO system change and another afterward. However, in the group of eight patients whose ECMO systems were not changed, only one Seraph 100 procedure was performed. All patients were treated with antimicrobials based on susceptibility testing and received corticosteroids according to the institution’s COVID guidelines.

**Figure 2:**
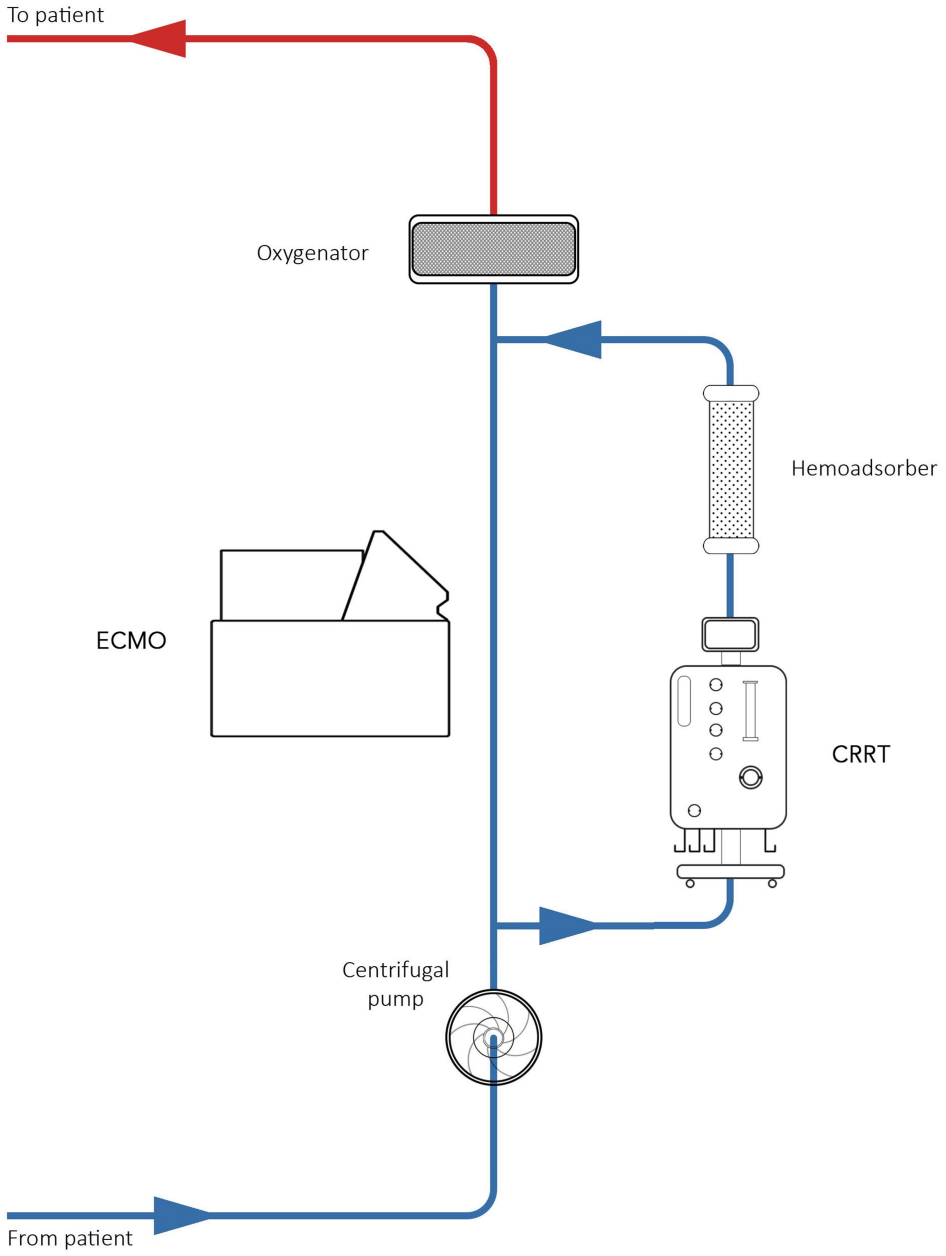
Schematic diagram of an ECMO circuit incorporating Seraph 100.

Due to technological issues, all patients with and without acute kidney injury (AKI) were treated with the Seraph 100 filter in series with the dialyzer (Figure 2). The blood from Seraph 100 was returned through the venous drainage section of the ECMO circuit. The blood flow rate was set to 150–200 mL/min. Seraph 100 treatments were prescribed for 4–6 h. The Maquet Cardiovascular Permanent Life Support System (Prolonged Life Support System; Maquet Inc., Rastatt, Germany) was used for ECMO in all patients. The PLS set consists of an oxygenator and a Rotaflow centrifugal pump, both incorporated into a tubing set with tip-to-tip Bioline (recombinant human albumin and heparin) coating.

The patients’ clinical parameters were extracted from ICU charts. The severity of organ dysfunction was assessed using the APACHE IV and SOFA scores (24). For each patient, complete clinical and laboratory examinations were performed upon admission to the ICU, at baseline, 24 h after the first hemadsorption procedure, and 3 days after the hemadsorption procedure. All blood samples were taken from the arterial line before entering the filter in the ECMO circuit.

The follow-up period lasted 28 days after the first hemadsorption procedure in the ICU or until death. We also recorded the antibiotics administered for any reason within 24 h before ECMO initiation, along with the reasons for ECMO support and the site of ECMO cannulation. The protocol, which did not deviate from standard practice implemented by clinicians, was approved by the hospital’s ethics committee (UHC Zagreb, Croatia) in accordance with the Declaration of Helsinki and its subsequent modifications. Data were managed in accordance with the patients’ written informed consent.

Statistical analysis was performed using SPSS Statistics 23 (IBM Corporation, Somers, NY, USA). The normality of data distribution was assessed using the Kolmogorov–Smirnov test. Categorical data were expressed as numbers and frequencies. Correlations were analyzed using Pearson’s test for normally distributed variables and Spearman’s rank correlation for non-normally distributed variables. Normally distributed variables were presented as means ± standard deviations, and Student’s *t*-test for independent samples was used for comparisons between the two groups. Non-normally distributed data were presented as medians and interquartile ranges, and the Mann–Whitney U-test was used for comparisons between the two groups. Categorical variables were compared using the *χ*^2^ test. Survival analysis was performed using Kaplan–Meier curves, with differences tested by the log-rank test, while hazard ratios were estimated using Cox proportional hazards regression. A *p*-value of <0.05 (two-sided tests) was considered statistically significant.

## Results

In total, 13 patients who required ECMO support were treated with Seraph 100. The median age was 49 years (age range: 33–68), while the BMI was 31.0, and 84.6% of the patients were men. The average ECMO flow in all patients was 3.7 L/min, with a target mean arterial pressure (MAP) maintained above 65 mmHg. The patients were on ECMO for an average of 14.5 days before experiencing a circuit-related infection.

Four patients required VA-ECMO, while the remaining nine patients required VV-ECMO (Table 1). No serious adverse events associated with Seraph treatment were observed, such as severe bleeding, thromboembolism, or electrolyte disorders.

**Table 1:** Demographic data and clinical parameters on the day of ECMO cannula-related infection diagnosis.

Variables	All patients (*N* = 13)
Age (years)	49 (33–68)
Sex (male) *N* (%)	11 (84.6)
BMI	31.0 ± 5.9
Comorbidities *N* (%)	
Diabetes	3 (23.1)
Hypertension	5 (38.5)
Hematological disease	3 (23.1)
Prior organ transplant	1 (7.7)
Prior cardiovascular event	3 (23.1)
Heart failure	3 (23.1)
Obesity	6 (46.1)
Days of ICU stay before ECMO	4.3 ± 0.6
Reason for ECMO *N* (%)	
Cardiac arrest	3 (23.1)
Acute myocardial infarction	1 (7.7)
ARDS	9 (69.2)
ECMO type *N* (%)	
VV	9 (69.2)
VA	4 (30.8)
Days on ECMO in ICU before cannula-related infection	14.5 ± 2.2
Glasgow coma score	8.6 ± 1.5
Vasoactive therapy Yes (*N* (%))	9 (69.2)
Vasoactive therapy dose mcg/kg/min	0.5 ± 0.3
Urinary output (ml/h)	75.7 ± 8.6
Acute kidney injury Yes (*N* (%))	4 (30.7)
Mechanical ventilation Yes (*N* (%))	13 (100)
APACHE IV	117.5 ± 27.5
SOFA score	10.3 ± 2.5

ECMO, extracorporeal membrane oxygenation; ICU, intensive care unit; BMI, body mass index; NIV, non-invasive mechanical ventilation; ARDS, acute respiratory distress syndrome; results are shown as mean +/− SD or median (interquartile range); *-chi square.

### Cannula-related infections and removal rates after Seraph 100 treatment

The most frequently isolated microorganisms were *Klebsiella pneumoniae* (38.4%), *Staphylococcus epidermidis.* (30.7%), *Acinetobacter baumannii* (30.7%), and *Candida (parapsilosis/glabrata)* (46.1%). Sterile blood cultures were achieved with Seraph 100 in 53.8% of all patients. Blood cultures after treatment with Seraph 100 were sterile for fungi (*Candida parapsilosis/glabrata*) in all patients, including both subgroups—those with and without ECMO system change due to cannula-related infections (Table 2). We found the same for *Stenotrophomonas malthophilia*. Blood cultures were sterile after Seraph treatment for *Klebsiella pneumoniae* in 60%, *Staphylococcus epidermidis in* 75%, and *Acinetobacter baumanii* in 50% of all patients. There was a higher percentage of sterile blood cultures for all microorganisms in the group in which the ECMO system was changed (80.0% vs. 37.5%). The percentage of sterile blood cultures for *Klebsiella pneumoniae* and *Acinetobacter baumanii* was higher in the patients who underwent ECMO system change than in those who did not (100% vs. 0%; 100% vs. 33%). In contrast, the percentage of sterile blood cultures for *Staphylococcus epidermidis* was higher in the patients without ECMO system change (50% vs. 100%). In total, nine of the 13 patients with circuit-related infections tested negative after treatment with Seraph, including five patients who did not have their cannulas changed.

**Table 2:** Removal of different microorganisms after Seraph 100 treatment in all patients and in subgroups with and without ECMO system change.

Microorganisms	Days before Seraph 100 treatment	Days after Seraph 100 treatment
All patients (*N*)		
*Klebsiella*	5	2
*Acinetobacter baumanii*	4	2
*Staphylococcus epidermidis*	4	1
*Stenotrophomonas malthophilia*	1	0
*Candida*	6	0
*Glabrata*	3	0
*Parapsilosis*	3	0
Patients with ECMO system change (*N*)		
*Klebsiella*	3	0
*Acinetobacter baumanii*	1	0
*Staphylococcus epidermidis*	2	1
*Stenotrophomonas malthophilia*	1	0
*Candida*	3	0
*Glabrata*	1	0
*Parapsilosis*	2	0
Patients without ECMO system change (*N*)		
*Klebsiella*	2	2
*Acinetobacter baumanii*	3	2
*Staphylococcus epidermidis*	2	0
*Stenotrophomonas malthophilia*	0	0
*Candida*	3	0
*Glabrata*	2	0
*Parapsilosis*	1	0

ECMO, extracorporeal membrane oxygenation.

### Variations in clinical and laboratory parameters in the patients treated with Seraph 100

We observed significant decreases in vasopressor doses and improvements in respiratory parameters on the first day after treatment with Seraph 100 (Table 3). No significant differences were observed in inflammatory parameters or lactate levels before and after treatment with Seraph 100, except for a decrease in white blood cell count following treatment. No differences were observed in the APACHE IV score before and after treatment, but there was a significant decrease in the SOFA score on the first day after Seraph 100 treatment. There were no differences in these variables between the patients with and without ECMO circuit change.

**Table 3:** Intra-group overtime variation in clinical parameters and laboratory data.

All patients	Days before Seraph 100 treatment	Days after Seraph 100 treatment	*p*-value
APACHE IV	117.5 ± 27.5	114.2 ± 26.9	0.12
SOFA score	10.3 ± 2.5	9.8 ± 2.6	**<0.001**
Vasoactive therapy (*N* (%))	9 (69.2)	5 (38.5)	0.11
Vasoactive dose mcg/kg/min	0.48 ± 0.01	0.25 ± 0.01	**0.04**
Lactate (mmol/l)	1.82 ± 0.3	1.72 ± 0.2	0.69
PEEP (cmH_2_O)	12.6 ± 1.8	9.7 ± 1.5	0.19
PaO_2_ (kPa)	9.3 ± 0.3	9.7 ± 0.3	0.37
FiO_2_ (%)	78.2 ± 13.9	63.3 ± 12.8	**0.03**
PaO_2_/FiO2	89.2 ± 20.1	114.9 ± 24.3	**<0.01**
PaCO_2_ (kPa)	6.7 ± 0.4	6.4 ± 0.4	0.66
Creatinine (umol/l)	178.2 ± 27.8	156.3 ± 24.8	0.21
Bilirubin (umol/l)	18 (6–31)	17 (6–30)	0.75
LDH (U/l)	521.3 ± 53.2	566.4 ± 91.1	0.35
Platelets (x10^9^/l)	155.8 ± 30.3	141.9 ± 28.7	0.19
D-dimers (mg/l)	3.3 ± 2.5	3.5 ± 1.4	0.12
White blood count (x10^9^/l)	15.3 ± 1.9	11.7 ± 1.6	**0.02**
Hematocrit (%)	32.1 ± 2.1	28.7 ± 1.8	0.16
Procalcitonin (mg/l)	5.2 ± 1.1	5.2 ± 1.3	0.87
C-reactive protein (mg/l)	161.3 ± 34.9	167.3 ± 35.2	0.37

ICU, intensive care unit; BMI, body mass index; NIV, non-invasive mechanical ventilation; ARDS, acute respiratory distress syndrome; LDH, lactate dehydrogenase; results are shown as mean +/− SD or median (interquartile range). Bold value indicates statistically significant.

### Survival of the patients treated with seraph 100

The calculated expected mortality rate based on the SAVE score for these patients was 56.0%, while ICU mortality among our patients treated with Seraph 100 was 53.8%. No differences were found for age and comorbidities between the patients who survived and those who did not after treatment with Seraph 100. All deceased patients were receiving higher doses of vasoactive therapy on the day when cannula-related infection was diagnosed compared to the survivors. There was no difference in the number of days on mechanical ventilation between the survivors and non-survivors; however, the non-survivors had higher SOFA scores than the survivors. Higher SOFA scores and vasoactive support were associated with increased mortality in the entire group (HR 2.24 [1.86, 2.64] and HR 1.82 [1.32, 2.28], respectively). By the end of the 28-day follow-up period, seven (53.8%) deaths occurred in the entire patient group. There was no difference in mean survival time between the patients with and without ECMO circuit change.

## Discussion

This is the first study to evaluate the reduction of cannula-related infections by Seraph 100 in COVID-19 patients on ECMO. The main finding of this study is that negative blood cultures after treatment with Seraph 100 were achieved in more than 50% of the patients. Our results also suggested that there was an association between Seraph 100 therapy and decreased doses of vasopressor support and improved SOFA scores.

The results are consistent with previous reports on normalization of hemodynamics and reduction of inflammatory biomarkers after Seraph 100 filter treatment (18, 21), as well as its beneficial effects in removing pathogens in septic patients (22, 23). However, this is the first study to describe the use of Seraph 100 in COVID-19 patients on ECMO for treating cannula-related infections.

The most frequently isolated microorganisms were *Klebsiella pneumoniae* (38.4%), *Staphylococcus* epidermidis (30.7%), *Acinetobacter baumannii* (30.7%), and *Candida (parapsilosis/glabrata)* (46.1%). Studies that reported cannula-related infections in patients supported by ECMO showed similar results to this study regarding rates of isolated *Staphylococcus epidermidis* (8). Patients with COVID-19, due to prolonged ICU stay, were susceptible to bacterial superinfections, primarily with *Acinetobacter, Klebsiella, and Pseudomonas* (25–28). Although these reports did not specifically address cannula-related infections in patients on ECMO, the rates of infection with *Acinetobacter* and *Klebsiella* were similar to those observed in this study. The rates of fungal cannula-related infections were significantly higher in this study than in previous studies evaluating cannula-related infections in patients supported by ECMO (8–10).

The Seraph 100® MicrobindAffinity Blood Filter (ExThera, Martinez, CA), which was evaluated in this study, had previously received a European Conformité Européene (CE) mark for sale in the European Economic Area and Emergency Use Authorization (EUA) from the Food and Drug Administration (FDA) for the treatment of severe COVID-19. The solid ultramolecular weight polyethylene (UHMWPE) beads used in Seraph are not spherical but have an irregular morphology. This surface texture increases the surface area of the adsorbent bed significantly compared to solid spherical beads. Seraph 100 presents all of its affinity ligands on the outside of the solid UHMWPE beads, allowing it to bind cellular and viral targets that are too large to fit within mesopores (29). *In vitro* studies have demonstrated that many bacteria are bound to the Seraph 100 adsorption media, allowing up to an 85% reduction in bacteria concentration (19). However, the first human study by Eden et al. was unable to demonstrate a reduction in bacterial positive blood cultures with the use of Seraph 100 (30). In contrasts, results from case reports involving bacterial removal through hemadsorption with Seraph 100 in septic patients on ECMO have been promising, as are the findings of our study (22, 23).

The removal of fungi by Seraph 100 has been reported in an *in vitro* study (19) but, to the best of our knowledge, has not yet been reported in any human study. The results from this study showed 100% sterile blood cultures from fungi (*Candida parapsilosis/glabrata*) after treatment with Seraph 100 in all patients, including both subgroups—patients with and without ECMO system change due to cannula-related infections. The percentage of sterile blood cultures for other microorganisms was not as high (*Klebsiella pneumoniae* (60%), *Staphylococcus epidermidis*. (75%), and *Acinetobacter baumannii* (50%)), although still significant. Changing cannulas and central lines on the ECMO system in cases of circuit-related infections is not feasible, according to some reports (31), due to the high risk of potentially lethal complications, limited vascular access, and associated cost. Nevertheless, the removal or early change of the infected parts of the circuit should always be discussed in cases of septic shock.

Although the study included a small number of patients, the results showed that the percentage of sterile blood cultures for all microorganisms was higher in the group where the ECMO system was changed compared to the group without system change (80.0% vs. 37.5%). The percentage of sterile blood cultures for *Klebsiella pneumoniae and Acinetobacter baumannii* was higher in the patients with ECMO system change than in those without ECMO system change (100% vs. 0%; 100% vs 33%). However, in the case of *Staphylococcus epidermidis* infections, this benefit was not observed; in fact, clearance rates were higher in the patients without ECMO system change (50% vs. 100%). Another factor that might have influenced the higher percentage of sterile blood cultures is that, in the group with the entire ECMO system everywhere, two Seraph 100 procedures were performed, before and after the system change. However, in the patients without a system change, only one Seraph 100 procedure was performed.

Improvement in hemodynamics after Seraph 100 treatment has been previously reported in COVID-19 patients and patients on ECMO without COVID-19 (18, 21–23, 32, 33). This study is the first to show similar results in COVID-19 patients supported by ECMO. A significant association of negative blood cultures after Seraph 100 treatment with better survival in linear regression analysis was not found, but as a 13-patient study, this study was not powered for such analysis. Nevertheless, the significant improvement in respiratory status, reduction in vasoactive support, and significant decrease in the SOFA score after the Seraph 100 procedure(s) are most probably direct consequences of pathogen removal from both the surface of the cannulas and from the bloodstream. The mortality rate of 57.2% reported in this study was higher compared to previous studies on cannula-related infections in patients on ECMO; however, these studies were not conducted on COVID-19 patients (5, 10).

The first limitation of the study is that it is a single-center report with a small sample size. This study is retrospective in design, which limits the generalizability of our results to a larger population. Secondly, a serious limitation of the study and interpreting Seraph 100’s efficacy is that pathogen levels were not measured due to financial and technical constraints. Therefore, our conclusions regarding the reduction of the inflammatory response are only speculative. Furthermore, our patient group consisted of a heterogeneous population with multiple indications for ECMO. As a small pilot study, both VV- and VA-ECMO patients were included, which is another limitation of this study. The inability to measure bacterial load before and after treatment with the Seraph filter is another limitation of the study, as such data would have potentially provided direct evidence of the filter’s effectiveness in reducing bacteriemia in our patients and strengthened the value of our results. The levels of endotoxins were not determined before and after the procedure, which limited our ability to assess the effect of Seraph on the removal of Gram-negative bacteria from the bloodstream. In addition, the cannulas were not sent for microbiological analysis after removal, so we cannot exclude the possibility that the sources of infection were unrelated to the cannulas. Furthermore, blood cultures can become normal with treatment even without hemoadsorption, and we did not include a matched cohort of ECMO patients with similar blood cultures who were treated without the filter, so our conclusions regarding the effectiveness of Seraph in reducing bacteriemia should be taken with caution.

To the best of our knowledge, this is the first study on COVID-19 ECMO patients treated with Seraph 100 for circuit-related infections that showed a significant percentage of negative blood cultures after hemadsorption, mostly pronounced for fungi. Although our other findings of decreased vasopressor doses, reduced inflammatory markers, and lower APACHE IV and SOFA scores after Seraph 100 treatment have been previously reported, the association between negative blood cultures and these clinical improvements is an intriguing finding that should be confirmed in multicenter prospective studies. To overcome these limitations, studies with improved patient stratification and a larger number of enrolled patients are needed.
